# Undocumented migrant women in Denmark have inadequate access to pregnancy screening and have a higher prevalence Hepatitis B virus infection compared to documented migrants in Denmark: a prevalence study

**DOI:** 10.1186/s12889-016-3096-8

**Published:** 2016-05-23

**Authors:** Annika Wendland, Boje K. Ehmsen, Vibeke Lenskjold, Birgitte S. Astrup, Marlene Mohr, Christopher J. Williams, Susan A. Cowan

**Affiliations:** Statens Serum Institut, Artillerivej 5, 2300 København S, Denmark; European Programme for Intervention Epidemiology Training (EPIET), European Centre for Disease Prevention and Control (ECDC), Stockholm, Sweden; Danish Research Centre for Migration, Ethnicity and Health (MESU), Øster Farimagsgade 5, Postboks 2099, 1014 København K, Denmark; Røde Kors Sundhedsklinikken, Blegdamsvej 27, 2100 København Ø, Denmark; ProVest Clinic, Fredericia, Denmark; Reden International, Colbjørnsensgade 12 st.tv, 1652 København V, Denmark; Public Health Wales, Temple of Peace and Health, Cathays Park, Cardiff, CF10 3AP UK

**Keywords:** Undocumented migrants, Pregnancy screening, HIV, Hepatitis B, Syphilis

## Abstract

**Background:**

Pregnant residents of Denmark are tested by their GP for current infections with Hepatitis B virus (HBV), HIV and syphilis through the Danish pregnancy screening programme to identify infections and initiate interventions to prevent mother-to-child transmission. Documented migrants (DM) have access to this screening but undocumented migrants (UM) do not, instead relying on ad-hoc care from clinics run by non-governmental organisations. We aimed to assess screening frequency in UM and to compare prevalence of infection in UM with DM.

**Methods:**

We obtained individual-level information on HBV, HIV and syphilis testing frequency and results for pregnant women attending three clinics specialising in care for UM between August 2011 and August 2014. We obtained aggregate data on the prevalence of the three infections for documented migrants from the Danish pregnancy screening programme and birth register between January 2011 and January 2014. Planned abortions were excluded from the study. We described demographic features of pregnant UM and estimated the screening frequency for HIV, HBV and syphilis. We compared prevalence of current infections in UM and DM by calculating standardised prevalence ratios (SPR).

**Results:**

The three UM clinics registered 219 pregnancies qualifying for screening. Overall 43, 58 and 60 % of pregnant UM had a test result recorded for HBV, Syphilis and HIV respectively, compared to >99 % in the general Danish population including DM.

The prevalence of HBV was higher in UM than in DM (SPR: 2.4; 95 % CI: 1.1–5.3). The SPR of 2 (95 % CI: 0.5–8.0) for HIV was not statistically significant, potentially due to small sample size of UM. None of the pregnant UM tested positive for Syphilis.

**Conclusions:**

Pregnant UM have a poorer chance of being tested for HIV, HBV and syphilis, despite having a higher prevalence of HBV than DM. We recommend giving systematic access to routine pregnancy screening to all UM to prevent mother-to-child transmission and to address the observed health care inequity.

## Background

Undocumented migrants (UM) are persons living in a country different to their country of origin with no official registration in their country of residence. The majority of member states of the United Nations have ratified treaties recognizing the right to equal and equitable access to health care for all persons, notwithstanding their legal standing within a government system [1]. Access to health care for UM however varies considerably between countries, and is generally lower than for legal residents [1–3]. Not having access to appropriate care can lead to severe outcomes of infections that could otherwise be managed or treated.

Screening of pregnant women for HIV, Hepatitis B (HBV) and syphilis is an effective method to detect infection in the mother. Simple and cost effective interventions are available to prevent mother-to-child transmission of these illnesses. If untreated, the risk of mother-to-child transmission of HIV during pregnancy, delivery or breastfeeding is 20–25 % and administration of antiretroviral drugs can reduce this risk to <1 % [4]. HBV infection of babies is associated with prematurity and lower birthweight, and 90 % of newborns infected with HBV develop chronic hepatitis if no vaccination is administered at birth [5]. Treatment with penicillin before the 12th week of pregnancy reduces the likelihood of complications due to syphilis from 20 to 70 to 2 % [6].

Denmark signed the UN treaties assuring equal and equitable access to health care. The Danish population of UM has increased from between 9000 and 21,000 persons in 2008 to 20,000–49,000 people in 2013 [7]. UM do not have the right to apply for a civil registration number (CPR number) entitling them to free access to health care services in Denmark. Their contacts with healthcare, including administered treatments, are not recorded in official databases.

The Danish pregnancy screening programme includes testing of all expecting mothers for HIV, HBV and syphilis. The implementation of universal pregnancy screening in Denmark was a consequence of inconsistent testing when targeting ‘at risk’ populations only. As a result, children were born without receiving appropriate interventions, resulting in mother-to-child transmission of preventable infectious diseases [8]. In 2013, between 99 and 100 % of the pregnant population were screened for those three pathogens and 0.26 % tested positive for HBV, 0.06 % tested positive for HIV and 0.005 % tested positive for syphilis. The majority of those infections were in documented migrants (DM) and were acquired outside of Denmark [9].

UM do not have access to the Danish pregnancy screening programme and instead rely on ad-hoc care from clinics run by non-governmental organisations (NGO clinics) specialising in UM health. In one of these clinics located in Copenhagen, a high proportion (12.9 %) of visits were related to pregnancy, childbearing and family planning [10]. We aimed to: 1) describe UM attending the clinics for a pregnancy, including gestational age at first visit, country of origin and pregnancy outcome; 2) estimate the proportion of pregnant UM attending NGO clinics, by region of origin, that are screened for HIV, HBV and syphilis; 3) estimate the number of pregnant UM and DM in Denmark testing positive for HIV, HBV and syphilis and compare the prevalences between the two groups, stratified by region of origin; and 4) identify if pregnant UM who tested positive for HIV, HBV and/or syphilis were followed up adequately to prevent mother-to-child transmission. Based on the study results we aimed to make recommendations to optimise screening and treatment of pregnant UM and their children, to prevent mother-to-child transmission of HIV, HBV and syphilis.

## Methods

### Sampling

We retrospectively identified pregnant UM residing in Denmark through three NGO clinics specializing in providing care to UM. All three clinics referred persons with CPR numbers to the Danish health care system, and therefore only registered UM in their own databases. We obtained information on females aged between 18 and 45 years at the time of data collection, who had visited the clinics between August 2011 and August 2014. Clinic A is located in Copenhagen and is open to all undocumented migrants. Note that for clinic A, we only extracted information until the end of May 2014. Clinic B is located in Jutland, the western part of Denmark and focuses on trafficked individuals, primarily sex workers. Clinic C is located in Copenhagen and has the same target population as clinic B.

Individual level information on DM was not available for the study. To estimate the proportion of pregnant DM screening positive, we obtained denominator data from the Danish Statistics Department on the number of children born in Denmark between 2011 and 2013, stratified by country of origin of the mother. Following a previously used approach [8], we considered the number of children born as a proxy for the number of pregnant women in Denmark with non-Danish origin. For the numerators, we obtained screening results for women from the Danish pregnancy screening database, housed at the Statens Serum Institut, which records all positive test results for HIV, HBV and syphilis. Only women who choose to keep their pregnancy are screened in the Danish pregnancy screening programme.

### Data collection

For UM, we extracted information on pregnancy status (pregnancy with no adverse outcome recorded, planned abortion, spontaneous abortion, post-natal visit), date of birth, date of first pregnancy related visit, country of origin, gestational week at first pregnancy visit, whether a specimen was collected for HIV, HBV and syphilis screening, and any test results recorded for those three illnesses. Additionally, we collected information on follow up done for women who had a positive test result recorded. Data was obtained from electronic patient management software, electronic and paper based pregnancy journals, electronic and paper based laboratory results and additional paper based patient records, as available in the clinics.

For DM, we extracted all births from 2011 to 2013 (3 years) by year of birth and mother’s country of origin from the Danish Statistics Department website. Additionally, we extracted all positive HIV, HBV and syphilis test results by country of origin of the mother from the Danish pregnancy screening database.

### Laboratory testing

We considered a positive screening result recorded in any of the data sources as a positive case. The tests used in the different clinics are listed in Table 1.

**Table 1:** Tests used for diagnosis of HIV, HBV and syphilis at the participating clinics and in the Danish pregnancy screening programme

Facility	HIV test used	HBV test used	Syphilis test used
Clinic A	Visual read qualitative immunoassay (Alere™ Determine™ HIV-1/2, Alere Medical Co. Ltd., Japan)	Hepatitis B surface antigen test (LIAISON® XL, DiaSorin S.p.A, Saluggia, Italy)	Point of care test (Core™ Syphilis, CORE Diagnostics, England), and confirmatory test in microbiological laboratory
Clinic B	HIV antibody test (ADVIA Centaur CP Immunoassay System, Siemens Healthcare GmbH, Germany)	Hepatitis B surface antigen test (ADVIA Centaur CP Immunoassay System, Siemens Healthcare GmbH, Germany)	Treponema antibody screening and Trepanmoa pallidum confirmatory test (ADVIA Centaur CP Immunoassay System, Siemens Healthcare GmbH, Germany)
Clinic C	Visual read qualitative immunoassay (Alere™ Determine™ HIV-1/2, Alere Medical Co. Ltd., Japan)	Hepatitis B surface antigen test (ADVIA Centaur XP Immunoassay System, Siemens Healthcare GmbH, Germany)	Point of care test (Core™ Syphilis, CORE Diagnostics, England), England and confirmatory test in microbiological laboratory
Danish pregnancy screening program	Tested at Danish blood banks, using a variety of automated diagnostic platforms		

### Exclusions

In the Danish pregnancy screening programme, women who wish to have an abortion are not offered routine pregnancy screening. We therefore excluded UM with a recorded planned abortion from the study population.

### Proportion of women screened

We defined a pregnancy as successfully screened if a negative or positive test result was recorded during the pregnancy with no subsequent note of test failure. We made note of specimens collected with no result recorded, but did not include these episodes in further analysis. For episodes with a specific reason given for not testing, we recorded the reason and classified the patient as not tested. Finally, if the notes (including all laboratory and patient records) contained no record of either testing or not testing, we considered the patient not screened for the purpose of this paper. To calculate screening frequency, we divided the number of women with a screening result recorded by the total number of women eligible for screening.

### Measuring prevalence of key infections

For UM, we calculated the prevalence for each illness by dividing the number of positive tests by the number of episodes with a screening result recorded (positive of negative), stratified by region of origin.

For DM, we divided the total number of pregnant women testing positive for HIV, HBV and syphilis through the Danish pregnancy screening programme, by the number of children born to women with non-Danish origin, stratified by region of origin.

### Follow up of UM women testing positive

We read medical records (paper based and electronic) of women with positive test results, and spoke to clinic staff to ascertain which follow up was done.

### Statistical analysis

We calculated median gestational age at first visit including the interquartile range (non-normal distribution of gestational age).

To estimate the standardised prevalence ratio (SPR) of UM vs DM for each infection, we first calculated the expected number of positive cases for our UM sample size, based on the observed prevalences in DM, by region of origin. We then added the number of expected positives from the different regions, to obtain the overall number of expected cases. Subsequently we compared the observed with the expected number of positives in UM and estimated the SPR and 95 % confidence intervals.

We used Stata statistical software, version 12.1 (StataCorp LP, 4905 Lakeway Drive, College Station, TX 77845, USA) and Statistical Analysis System (SAS) software, version 9.4 (SAS Institute Inc, 100 SAS Campus Drive, NC 27513–2414, USA).

## Results

### Description of population

**Fig. 1:**
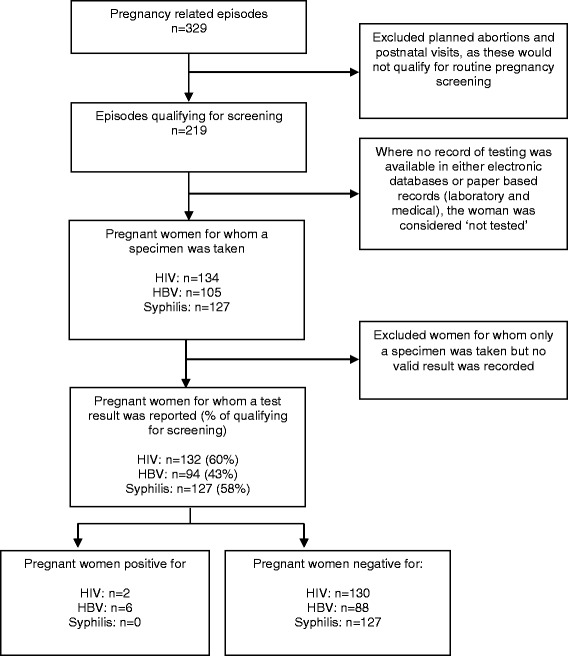
Selection of pregnant undocumented migrant women at 3 NGO clinics, for the study of screening patterns and prevalence of HIV, HBV and Syphilis, Denmark, August 2011 to August 2014

Between January 2011 and May 2014, UM visited the three clinics for 329 pregnancy related episodes. Of those, 201 (61 %) were for pregnancies with no recorded complications and 18 (5 %) resulted in spontaneous abortions. Planned abortions were performed for 77 (23 %) of the episodes, and 33 (10 %) related to post-natal visits (Fig. 1, Table 2). We considered episodes qualifying for screening if women were pregnant at the time of the visit and no planned abortion was recorded in the notes.

**Table 2:** Pregnancy status and country of origin for undocumented migrants presenting to three NGO health clinics in Denmark, August 2011 to August 2014

		Clinic A		Clinic B		Clinic C		Total	
		Number	%	Number	%	Number	%	Number	%
Total		267		38		24		329	
Pregnancy status	Normal pregnancy	181	68 %	14	37 %	6	25 %	201	61 %
	Spontaneous abortion	15	6 %	2	5 %	1	4 %	18	5 %
	Planned abortion	41	15 %	19	50 %	17	71 %	77	23 %
	Postnatal visit	30	11 %	3	8 %	0	0 %	33	10 %
Region of origin	Eastern Europe	41	15 %	17	45 %	5	21 %	63	19 %
	Indian subcontinent	43	16 %	0	0 %	0	0 %	43	13 %
	Middle East/ North Africa	45	17 %	3	8 %	0	0 %	48	15 %
	North America	1	0 %	0	0 %	0	0 %	1	0 %
	South- and Middle America	12	4 %	6	16 %	0	0 %	18	5 %
	South-East Asia	50	19 %	1	3 %	2	8 %	53	16 %
	Sub-Saharan Africa	73	27 %	11	29 %	14	58 %	98	30 %
	Western Europe	1	0 %	0	0 %	0	0 %	1	0 %
	unknown	1	0 %	0	0 %	3	13 %	4	1 %

Gestational age at first visit was available for clinic A and B. Excluding visits related to planned abortions, the median gestational age at first visit was 12 weeks (IQR: 8 to 21 weeks). Women presented for the first time as late as week 41 of their pregnancy. Of 183 pregnancy related visits, 97 (53 %) occurred after week 10 of pregnancy.

Planned abortions were more common at sex worker clinics (B and C) than general UM clinic (A) (50–71 % vs 15 % of episodes). Sex worker clinic B had the highest proportion of Eastern European women (45 %), sex worker clinic C the highest proportion of Sub-Saharan African women (58 %), whereas general UM clinic A had a more even distribution of countries of origin with the most represented group coming from Sub-Saharan Africa (27 %) (Table 2).

### Proportion of women screened

Overall, 219 episodes qualified for screening (Fig. 1).

For HIV, 134 specimens were taken and 132 (60 %) had a screening result recorded (Fig. 1). Screening completeness ranged from 51 to 73 %. It was highest in women from the Middle East and North African countries and lowest in women from the Indian subcontinent and of Sub-Saharan origin (Table 3).

**Table 3:** Number and proportion of pregnant undocumented migrants (UM) out of total number of episodes eligible for screening, presenting to three NGO health clinics in Denmark, August 2011 to August 2014

	Pregnant UM qualifying for screening	UM seen at 3 clinics, for whom test result was recorded					
		HIV		HBV		Syphilis	
Subregion of origin	Number	Number	%	Number	%	Number	%
Eastern Europe	35	20	57 %	16	46 %	18	51 %
Indian subcontinent	37	19	51 %	10	27 %	18	49 %
Middle East/ North Africa	37	27	73 %	20	54 %	26	70 %
South- and Middle America	11	7	64 %	6	55 %	7	64 %
South-East Asia	36	25	69 %	18	50 %	25	69 %
Sub-Saharan Africa	60	32	53 %	22	37 %	31	52 %
Other	3	2	67 %	2	67 %	2	67 %
Total	219	132	60 %	94	43 %	127	58 %

For HBV, 105 specimens were collected and screening results were available for 94 (43 %) of them (Fig. 1). Screening completeness ranged from 27 to 55 % (excluding “Other”) and was highest in South- and Middle American women, and lowest in women from the Indian subcontinent (Table 3).

Syphilis specimens were taken for 127 episodes (58 %) and all had a screening result recorded (Fig. 1). Between 49 and 70 % of women were screened, with completeness highest in women from Middle Eastern and North African (MENA) countries and lowest in women from the Indian subcontinent (Table 3).

### Reasons for not screening

According to the medical records sighted (only available for clinic A and B), 47 of 78 women who were not screened had no reason recorded. Of those with a reason recorded, 11 were lost to follow up, nine had left the country, and seven were referred to a specialist clinic. The remaining four were not tested because they either declined the test, the test was unavailable at the time of visit, the consultation was unrelated to pregnancy or a previous positive test was done elsewhere prior to visit.

### Prevalence of HIV, HBV and syphilis

Of the 132 specimens with a screening result for HIV, two tested positive. Both cases attended clinic A. Extrapolating from the prevalence observed in DM, we would have expected to identify one positive case of HIV for our sample size. The standardized prevalence ratio (SPR) indicated double the prevalence of HIV in UM compared to DM, but the difference was statistically not significant. For HBV, six of 92 specimens tested positive, compared to an expected 2.51 cases testing positive. The SPR of 2.4 (95 % CI 1.1–5.3) indicates a significantly higher prevalence of HBV in UM compared to DM. Five of the specimens tested positive at clinic A and the sixth specimen originated from clinic B. None of the pregnant UM tested positive for syphilis (Table 4).

**Table 4:** Comparison of HIV, HBV and syphilis in documented vs undocumented migrants presenting to three NGO health clinics in Denmark, August 2011 to August 2014

		Documented migrants (DM)^a^			Undocumented migrants (UM)				UM vs DM	
		Total Population	Observed cases	Cases per 1000	Sampled Population	Observed cases	Expected cases	Cases per 1000	SPR^b^	95 % CI
HIV	Eastern Europe	7202	11	1.5	20	0	0.03	-		
	Indian subcontinent	1864	1	0.5	19	0	0.01	-		
	Middle East/ North Africa	6390	1	0.2	27	0	0.00	-		
	South- and Middle America	759	3	4.0	7	0	0.03	-		
	South-East Asia	3066	8	2.6	25	0	0.07	-		
	Sub-Saharan Africa	2379	64	26.9	32	2	0.86	62.5		
	Total	21,660	88	4.1	130	2	1.00	15.4	2.0	0.5–8.0
HBV	Eastern Europe	7202	82	11.4	16	1	0.18	62.5		
	Indian subcontinent	1864	16	8.6	10	0	0.09	-		
	Middle East/ North Africa	6390	79	12.4	20	1	0.25	50.0		
	South- and Middle America	759	3	4.0	6	0	0.02	-		
	South-East Asia	3066	192	62.6	18	0	1.13	-		
	Sub-Saharan Africa	2379	91	38.3	22	4	0.84	181.8		
	Total	21,660	463	21.4	92	6	2.51	65.2	2.4	1.1–5.3
Syphilis	Eastern Europe	7202	51	7.1	18	0	0.13	-		
	Indian subcontinent	1864	2	1.1	18	0	0.02	-		
	Middle East/ North Africa	6390	16	2.5	26	0	0.07	-		
	South- and Middle America	759	12	15.8	7	0	0.11	-		
	South-East Asia	3066	24	7.8	25	0	0.20	-		
	Sub-Saharan Africa	2379	38	16.0	31	0	0.50	-		
	Total	21,660	143	6.6	125	0	1.01	-	-	-

^a^documented migrants were considered the standard population composition for migrants in Denmark and used to calculate the expected number of undocumented migrant cases

^b^ *SPR* standardised prevalence ratio

### Follow up of UM women testing positive

At one clinic, two of the women with positive tests for HBV were initially falsely classified as negative, and follow up had not been initiated until we conducted this study. One woman could be contacted. She had obtained a Danish residency permit and we referred her to her general practitioner for appropriate follow up of her and her child. The other woman was lost to follow up and no information was available on whether vaccinations were administered to her child.

Of the 4 pregnancies correctly diagnosed as HBV positive, one attended the clinic only once and returned to her home country. Information was provided to her over the phone regarding her test result and the consequences, and she was advised to enrol in the pregnancy care programme of her home country. One woman had full records of follow up and vaccination of the child at the NGO clinic. One woman had a spontaneous abortion at 3 months and the fourth woman had no follow up recorded in the notes.

The women testing positive for HIV were both referred to venereal departments in Danish hospitals for follow up.

## Discussion

### Late presentation

Just over half of the women visited the clinic for the first time after week 10 of their pregnancy. The recommendation for the first pregnancy related visit in Denmark is between gestational week 6 and 10. Late presentation for pregnancy related care may lead to delayed interventions for the prevention of mother-to-child transmission of infectious diseases. Possible barriers to accessing health services include limited medical rights, fear of being reported to the police, poor language skills and lack of knowledge about the healthcare system and informal networks of healthcare professionals [11].

### Abortions

A large proportion (50–71 %) of women attending the two sex worker clinics requested an abortion, suggesting a high level of unplanned pregnancies. Two studies from Switzerland showed that UM in general reported a high proportion of unplanned pregnancies (75 to 83 %) [12, 13] but we are unable to identify how many pregnancies beyond those resulting in an abortion were unplanned in our study. The information collected in this study did not allow distinguishing if women attending sex worker clinics fell pregnant to their regular partners or clients. Sex workers may use condoms consistently with their clients but may have unprotected sex with their partners, as a symbol of a more intimate relationship [14–16]. The high level of planned abortions indicates a need for better access to contraceptives and potentially a need for family planning services for UM, particularly for women working in the sex industry.

### Screening and prevalence

#### HIV

Only 60 % of women eligible for screening had a HIV test result recorded, compared to 99.9 % of the Danish population with legal residency status [9]. Screening was amongst the lowest in women of Sub-Saharan African origin, a concerning result, as prevalence of HIV is expected to be highest in that population. The observed number of cases in the UM population was two times higher than for DM, and the two cases detected were in Sub- Saharan African women attending clinic A, which is not a specialised sex worker clinic. The result was not statistically significant and the power of the study was too small to observe significant differences between UM and DM.

Early detection and treatment of pregnant women infected with HIV increases the chances of favourable outcomes for the child. Controlling the viral load will reduce the risk of intrauterine and post-natal transmission of HIV to the child. Late presentation during pregnancy may not allow for the reduction of the viral load to satisfactory level in time for the birth and increases the risk of infection in the child [4].

#### HBV

We observed the lowest percentage of recorded screening results for HBV: overall, only 43 % of pregnant UM had a screening result recorded, compared to 99.9 % of pregnant women with legal residency in Denmark [9]. Screening for HBV required sending of specimens to the laboratory, as there is currently no point of care test approved in Denmark. It is therefore a less convenient test to conduct and increases chances of either not testing or not documenting a test result in the patient’s records.

Our study results showed a 2.4 times higher prevalence of women testing positive for HBV during pregnancy if they are UM compared to DM.

A study published in 2015 found that 6 % of migrants – 94.2 % of which were undocumented - screened for HBV were found to be HBV surface antigen positive [17]. This is comparable to our findings, where we found a HBV prevalence of 6.1 % in our UM population.

WHO has classified the regions in the world by low, intermediate and high endemicity of HBV. Sub-Saharan African and South-East Asian countries fall into the high-endemicity areas, with prevalence of HBV >8 %. Eastern Europe, the Indian subcontinent, some South- and Middle American and MENA countries are classified as intermediate endemicity with prevalences of 2–7 % [18]. In these populations, screening would be particularly important, but in our study, only 37–50 % of women from high prevalence regions and 27–55 % of women from intermediate prevalence regions had a record of screening. Given the potential high prevalence of HBV in UM coming from these regions, low screening levels create a real risk that babies are born to undiagnosed HBV positive mothers, potentially missing the opportunity of vaccination to prevent HBV infection.

Women positive for HBV with high viral loads are more likely to transmit HBV to their children, even if the children receive the recommended vaccination course from birth. Detection of the illness during pregnancy allows for the administration of antivirals, reducing the risk of infection in the child which often leads to the development of a chronic HBV infection [19].

#### Syphilis

For syphilis, 58 % of UM women had a recorded screening result, compared to 99.7 % in pregnant women with documented residence in Denmark. We expected to detect approximately one case of syphilis given the population size and region of origin, but none of the women tested positive. Given the rarity of syphilis infection, the power of this study is likely too small to detect any significant differences in syphilis prevalence between UM and DM.

A concerning aspect for the management of syphilis in pregnant women is the relatively late presentation for first pregnancy related health checks, with 42 % of UM in this study attending a clinic after week 12 of their pregnancy. For syphilis, antibiotic treatment should be initiated by week 12 of pregnancy to prevent complications including spontaneous abortions, premature delivery, stillbirths, perinatal death or serious sequelae in children born with congenital syphilis [6].

### Follow up

Two women who tested positive for HBV by HBsAg test were falsely noted as negative in the patient notes. We tried to contact these women and were able to reach one, who was not aware that she had a chronic infection with HBV and whose child did not receive any HBV vaccinations. The second woman was lost to follow up.

The clinics providing health services to pregnant UM all cited similar challenges in regards to follow up and adequate treatment of pregnant women. If women did not return to obtain their screening results, a referral could be made to initiate treatment or plan for vaccination, putting the child (and potentially sexual partners) at risk of infection. Additionally, the clinics were operated by many health care staff who mostly work on a volunteer basis. The clinics provided services often supplied by specialists in the regular healthcare system, and even though volunteers were highly qualified, they might not have the familiarity with all the situations they encountered at the clinics, compared to someone who routinely performs the work. It was difficult to maintain consistent training on standard operating procedures for all volunteers working at the clinics. At the time of the investigation, the three clinics were among very few options available to UM to obtain basic perinatal services.

### Bias and limitations

As the population studied consisted of undocumented individuals, we had to use a convenience sample of all UM presenting to one of three NGO clinics during the study period. We had no information on the proportion of pregnant UM who did present to a clinic, and therefore are unable to tell how many additional women missed screening. We could not tell if the women presenting to a health clinic were representative of the pregnant UM in Denmark, and if they were more or less healthy. It is possible that entire ethnic groups did not attend a health clinic if there were barriers to seeking health care that we are unable to quantify.

We collected information from clinics with different population profiles: clinic A may be more representative of a general UM population, whilst women attending clinics B and C were almost exclusively sex workers. Sex workers were at higher risk of infection with HIV, HBV and syphilis and could lead to an overestimation of the illness. We did not however see any HIV infection in any of the sex worker clinics, and did not observe a significant difference in HBV prevalence between the sex worker clinics and the general UM clinic.

We could only collect retrospective data and were limited to information collected in the clinic records. We were able to identify reasons for not testing for 30 of 77 women who were eligible for screening but had no test result recorded. For the remaining 61 % we were unable to identify if women with no record of testing available were truly not tested, or if this is a record keeping issue. It is possible that negative test results are less often recorded, which could lead to an overestimation of prevalence, or that additional positives have been missed by failure to test, which could lead to an underestimation of prevalence. One woman was reported to have tested positive for HBV elsewhere but no laboratory test result was available in the clinic records. This woman was therefore excluded from the prevalence analysis, but had she been included, the prevalence of HBV in UM would have been even higher.

## Conclusions

Our results show much lower access to pregnancy screening for UM compared to the Danish population. Additionally, despite the small sample size, results show a higher prevalence of HBV infection in pregnant UM compared to DM after adjustment for region or origin, emphasising that screening of this population is essential to protect the unborn child.

Potential perceived risk by UM of utilizing systematic screening within the Danish health care system may include the fear of having personal data recorded in an official register. These data could be used inappropriately for the seizure and expulsion of UM, who may therefore choose not to utilise screening. Given the high proportion of pregnancies in UM women seeking healthcare, we do believe however, that if a safe and accessible service for screening were available, pregnant UM would choose to use this to ensure good health during their pregnancy and favourable outcomes for their children.

Providing free and systematic access to pregnancy screening is in line with international treaties ratified by the Danish government, agreeing to provide equal and equitable access to health care for UM, including pre- and postnatal care and right to health for children of UM [1]. Additionally, preventative measures have been shown to be cost effective [20–22]. Currently, the clinics providing pregnancy care to UM operate on budgets reliant on fund raising and donations. This poses challenges when aiming to ensure continuous management of chronic infections such as HIV and HBV. Interruptions in treatment can have detrimental consequences for patients.

To our knowledge, this study is the first attempt to quantify access of UM to pregnancy screening in Denmark and to estimate the prevalence of HIV, HBV and syphilis in this population. Based on our results, we believe that including pregnant UM women in routine pregnancy screening programs and offering them perinatal care will lead to earlier presentation to health care services and improve screening coverage for preventable infectious diseases to protect the child from vertical transmission with HIV, HBV and syphilis. We recommend giving systematic access to routine pregnancy screening to all UM, integrated into the regular healthcare system, to protect newborn babies from infection with preventable diseases and to address the observed health care inequity.

## Abbreviations

DM: documented migrants
HBV: Hepatitis B virus
HIV: human immunodeficiency virus
IQR: inter quartile range
MENA: Middle East and North Africa
SPR: standardized prevalence ratio
UM: undocumented migrant
